# Clinical profile of HIV/AIDS patients admitted to a tertiary outpatient clinic in Istanbul, Turkey

**DOI:** 10.1186/1758-2652-13-S4-P220

**Published:** 2010-11-08

**Authors:** O Aydin Altuntas, H Kumbasar Karaosmanoglu, R Korkusuz, O Nazlican

**Affiliations:** 1 Haseki Training and Research Hospital, Infectious Diseases and Clinical Microbiology, Istanbul, Turkey; 2 Haseki Traininig and Research Hospital, Infectious Diseases and Clinical Microbiology, Istanbul, Turkey

## Purpose of the study

The clinical course of HIV/AIDS and pattern of opportunistic infections vary from patient to patient and from country to country. Hence, we aimed to study clinical presentation of HIV/AIDS patients according to their first admission sequence to our outpatient clinic.

## Methods

Between the term of January 2006 and May 2010, 156 HIV infected patients were admitted to our center. Clinical data about patients were collected retrospectively from standardized HIV/AIDS forms filled at the time of admission.

## Results

Out of 156 patients, 73 (47%) were admitted with obvious clinical signs and symptoms, whereas 83 (53%) had no signs and symptoms, and they were diagnosed through screens or check-up tests. The most frequent clinical symptoms on first admission were mucocutaneous manifestations of HIV infection (13,5%), weight loss (8%), persistent generalized lymphadenopathy (4,5%), pulmonary tuberculosis (4%), malignancies (4%), *Pneumocystis jirovecii* pneumoniae (2,5%) and chronic fever (2,5%). Screening tests were conducted most frequently during blood donations (12%), before surgeries (11,5%) and check-ups (7,7%). Among these cases 11,5% were diagnosed by volunteered HIV tests.

## Conclusions

Turkey is among low prevalence countries in Europe for HIV/AIDS. Clinical profile of our patients is similar to the developed countries. More than half of the patients diagnosed through screens or check-up tests, which give satisfactory results at the early stages of the disease. Nevertheless, screening tests and encouragement of volunteered HIV tests for individuals within high risk groups may increase the number of early diagnosed cases and help to reduce the spreading of the virus.

